# Plane crash in Lake Victoria, Tanzania: What it says of air safety in Africa

**DOI:** 10.1002/puh2.107

**Published:** 2023-07-17

**Authors:** Innocent Kitandu Paul, Jonaviva Anthony Thomas, Zenno Joseph Kavishe, Alfred Jubilate, Goodluck Nchasi

**Affiliations:** ^1^ Faculty of Medicine Catholic University of Health and Allied Sciences Mwanza Tanzania; ^2^ Faculty of Medicine Kilimanjaro Christian Medical University College Moshi Tanzania; ^3^ Faculty of Medicine Muhimbili University of Health and Allied Sciences Dar es Salaam Tanzania

Dear Editor,

Achieving aviation safety is still a challenge in the African region. The field does not receive much attention before the occurrence of accidents, resulting in tragic outcomes such as high numbers of deaths and injuries. On 6 November 2022, a Tanzanian Precision Air company aircraft crashed in Lake Victoria following the pilots’ attempt to land it at Bukoba Airport. The accident resulted in 19 deaths out of the 43 passengers and crew onboard [1]. The main cause of the crash was attributed to a storm and bad weather conditions, which forced the pilots to reroute the aircraft and land it in the lake. Fishermen who were near the area responded immediately and were able to rescue the survivors of the crash [1]. Although the African aviation field lacks enough studies and research, one study revealed a total of 132 air accidents in the region from 2004 to 2013. It showed that the most common causes of these accidents were attributed to technical failure, pilot error, and the weather [2]. This article aims to explore the significant challenges that Tanzania faces concerning aviation safety and its relation to the African region as a whole.

The Bukoba Airport has seen increased utilization in recent years for residents of the Western Lake Victoria zone for regular flying and landing activities [3]. Inadequate aviation infrastructure has affected the activities at the airport and is seen as one of the major causes of the recent crash. According to the Tanzania Airports Authorities, the airstrip was 30 m wide and upgraded from 1200 to 1500 m. However, the airport still maintains a small parking area for the planes and is too closely situated to the runway making it difficult for airplanes to take off and land [4].

Climatic challenges, with the mostly cloudy and humid climate in the region throughout the year, create a challenge in maintaining the gravel runway, creating a dangerous environment for flying and landing activities. Moreover, the airport lacks traffic lights used to lead the pilot, making landing during humid weather difficult. The government and Tanzania Airports Authority (TAA) have attributed bad weather as the main cause of the tragic accident. Weather in the African continent is good for aviation activities but it is still included as one of the causes for accidents that occur in the region [2].

With poor utilization of rescue facilities [5], the TAA has reported that Bukoba Airport's Rescue and Fire Fighting Services (RFFS) station had all important facilities, including communication facilities and rescue equipment [4]. However, upon the tragedy, the team did not play a role in saving the lives of the deceased. Instead, it was the local fishermen who were near the area that played a larger role in the rescue of passengers with significant assistance from the flight's cabin crew. It has been a huge stumbling block for the development of many African countries not to strengthen preventive measures, property maintenance, or investment in early preparations leading to preventable but ultimately fatal consequences.

The other challenge is the purchase of used planes from manufacturing companies. Many African countries are not yet independent in constructing their aircraft and tools; instead, they purchase second‐hand used planes. For example, the plane that crashed in Lake Victoria was an ATR 42‐500 that was manufactured and assembled in 1984 [6]. It is possible the aircraft itself had unknown technical problems, and perhaps the pilots tried their level best to contain it and in the end land it in the lake about 100 m from the airport. The investigators from France and the company manufacturing the planes are in the country at the moment investigating the case.

Through the Ministries of Transportation, Security, and Health, the TAA, and the Precision Air Company, the government should amend policies and rules to govern and ensure safety in aviation activities by adopting the safety guidelines set by the International Civil Aviation Organization (ICAO) that govern the aviation field worldwide, to avoid and prevent unforeseen tragedies. The government should also initiate an immediate domestic investigation of the recent accident with the help of foreign expert investigators. Inspection of all homeland airports should be conducted to reveal discrepancies and prompt the Airports Authority to resolve them immediately. In addition, to improve the standards of aviation services in African countries, authorities should initiate and promote rescue training, install reliable communication facilities at the terminals, and ensure the presence of rescue teams and facilities at airports at all times. Moreover, aviation authorities in Africa should enact common policies and regulations that will promote and govern aviation activities in the region. Furthermore, policies on environmental conversation must be established and followed to minimize environmental hazards during aviation activities [7, 8]. While regular activities continue as usual at Bukoba Airport, weather forecasting and broadcasting need to be communicated to the authorities early to facilitate accurate planning of flying and landing activities to avoid air accidents. Furthermore, infrastructure at the airport should also be improved to allow the terminal to operate safely without fear of further unpredicted losses and fatality.

Lastly, to achieve aviation safety in the African continent is truly a difficult challenge that demands special attention and integral interventions from the authorities and prominent stakeholders. The lack of enough data in the field provokes the need for more research in this particular field to reveal further challenges facing it, and subsequently suggest ways to overcome them, rather than focusing only on tertiary interventions upon occurrence of fatal accidents. Nonetheless, to successfully attain aviation activities that are safe and sustainable, upgrading the aviation education system, advancement in technology, and improvement of infrastructures are highly required.

## AUTHOR CONTRIBUTIONS

*Conceptualization; writing—original draft; reviewing and editing*: Innocent Kitandu Paul. *Conceptualization; writing—original draft*: Jonaviva Anthony Thomas. *Reviewing and editing*: Zenno Joseph Kavishe and Alfred Jubilate. *Reviewing and editing the final draft*: Goodluck Nchasi.

## CONFLICT OF INTEREST STATEMENT

Authors declare no conflicts of interest.

## FUNDING INFORMATION

This work did not receive any funding.

## PATIENT CONSENT STATEMENT

No patient or patient data were utilized.

## CLINICAL TRIAL REGISTRATION

This is not clinical trial study.
